# Supporting Health-Care Workers and Patients in Quarantine Wards: Evidence From a Survey of Frontline Health-Care Workers and Inpatients With COVID-19 in Wuhan, China

**DOI:** 10.3389/fpubh.2021.705354

**Published:** 2021-10-18

**Authors:** Ting Zhou, Ruiyuan Guan, Susan L. Rosenthal, Scott Moerdler, Ziqi Guan, Liqun Sun

**Affiliations:** ^1^Department of Medical Psychology, School of Health Humanities, Peking University, Beijing, China; ^2^Department of Pediatrics, Columbia University Irving Medical Center-Vagelos College of Physicians and Surgeons, New York, NY, United States; ^3^Division of Pediatric Hematology Oncology, Rutgers Cancer Institute of New Jersey, New Brunswick, NJ, United States; ^4^Department of Psychology, University of Minnesota, Minneapolis, MN, United States; ^5^Intensive Care Unit, The Second Affiliated Hospital of Nanjing Medical University, Nanjing, China

**Keywords:** psychological symptoms, psychological stressors, coping resource, frontline health-care workers, hospitalized patients with COVID-19

## Abstract

**Objective:** Frontline health-care workers and patients with COVID-19 have been identified as high-risk groups for psychological problems. Experience of working or staying in quarantine wards generated psychological stressors for health-care workers and patients with COVID-19. The present study aimed to investigate the psychological symptoms of hospitalized patients with COVID-19 and the health-care workers treating them during the outbreak period, examine the effects of psychological stressors on mental health in both populations and perceived coping resources for both sides.

**Methods:** Three hundred and eleven health-care workers working in a COVID-19 designated hospital in Wuhan, China, and 148 hospitalized patients with COVID-19 in the same hospital participated in this cross-sectional survey conducted in February 2020. Psychological symptoms, psychological stressors, and perceived coping resources were reported by both groups.

**Results:** Thirty-three percent of health-care workers and 35.2% of patients with COVID-19 had significant psychological symptoms that were indicative of a high risk for psychological disorders. Pandemic-related psychological stressors contributed to psychological symptoms for both populations. Concern about patients was one aspect of psychological stressors of frontline health-care workers and both groups perceived support from the opposite side as an important external coping resource.

**Conclusion:** The results shed light on the need to provide psychological support to both frontline health-care workers and patients with COVID-19 and suggest enhancing the treatment alliance might be effective to improve mental health for both populations during the crisis.

## Introduction

The pandemic of COVID-19 has lasted for more than 1 year and continues to affect people around the world. This pandemic is not only a threat to the lives of people but also a great challenge to the mental health of people.

Health-care workers and patients with COVID-19 are heavily involved in the crisis and have been identified as high-risk populations for developing psychological problems (1). Understanding the mental health status of the health-care workers and patients during the peak period of the epidemic is essential to provide psychological support and interventions.

The psychological impact of frontline health-care workers has been reported during previous epidemics and the current COVID-19. As found by most studies, health-care workers experienced heightened levels of psychological symptoms including anxiety, depression, post-traumatic stress disorder (PTSD) during the outbreak of COVID-19 (2–5). Similar to situations in previous major epidemics, worries of being infected (6–8), concerns about the health conditions of the families (9, 10), frustration, and discomfort caused by protective measures (11) as well as difficulties in dealing with patients with emotional problems (12) have been considered as major psychological stressors for frontline health-care workers. Although the psychological stressors have been identified, how they contribute to the mental health status of frontline health-care workers has been less studied.

Studies focusing on the psychological experience of patients with COVID-19 are relatively fewer, especially during the period of hospitalization. As informed by qualitative research, patients reported their experience as “living in limbo” and because of the high mortality and uncertainty about the prognosis, participants were trapped between life and death (13). Therefore, the pressure of survival threat is an important psychological stressor for inpatients with COVID-19. Loneliness or lack of social support is also considered an important psychological stressor in COVID wards (9, 13, 14). Worries about the health conditions of family members and guilty for not being able to take care of them were also reported (15). Some patients also reported worries about being rejected and related to COVID-19 stigma (13). In the face of such psychological stressors, the mental health of patients with COVID-19 is treated. As indicated by the results of a few studies available, patients reported heightened levels of anxiety, depression, and insomnia (14, 16–18), and the symptoms last even after they have been discharged from the hospital and recovered (19, 20). Given the prevalence and significance of psychological problems in patients with COVID-19, it is important to identify the salient stressors in this population and find out the preference of psychological service of patients during the hospital stay.

It is important to note that previous studies focusing on frontline health-care workers and patients were usually carried out separately in different environments. Stressors and psychological reactions of frontline health-care workers and patients for whom they are providing treatment have been less examined simultaneously. Health-care workers and patients are two sides of therapeutic relationships. Both sides have the potential to influence emotions, cognitions, and behaviors of each other in a reciprocal way (21, 22). Furthermore, the quarantine ward is a specific environment where both health-care workers and patients face the threat of infectious disease and personal social support is limited for both populations. Investigating the psychological responses of patients in the crisis of COVID-19 is important to gain a better understanding of the situation of the health-care workers, and the reverse is also true. Furthermore, if the health-care workers and patients are considered as components of an interpersonal system (23), it is vital to take integrated psychological support strategies for both sides.

It has been widely accepted that the perception of coping resources plays an important role in the success of stress coping (24). Coping resources comprise a wide variety of behaviors (internal resources) and social networks (external resources) that aid the individual in dealing with stresses in life (25). Previous studies have shown that effective external coping resources for both health-care workers and patients are individual social support (26, 27). For the health-care workers, organizational support was positively associated with the mental health of the frontline health-care workers during major epidemics such as severe acute respiratory syndrome (SARS) and COVID-19 (28, 29). In addition, in the environment of COVID wards, the dyadic support between health-care workers and patients might be particularly important. Furthermore, with the development of mobile internet technology, various mental health services including online self-help psychological programs and virtual psychological counseling by telephone and internet were provided to the public in response to the COVID-19 outbreak. The online psychological services could be potential external resources for the frontline health-care workers and patients with COVID-19. However, the demands and preferences of these mental health services among health-care workers and patients were largely unknown.

Thus, the present study extends previous work by examining the experiences of frontline health-care workers and patients with COVID-19 within the same hospital environment during the rapid growth stage of the COVID-19 outbreak^1^. Specifically, we sought to (1) describe the psychological reactions of the frontline health-care workers and patients in the same COVID wards environment; (2) examine effects of the main psychological stressors on psychological symptoms for frontline health-care workers and patients with COVID-19; (3) identify perceived helpful external coping resources for frontline health-care workers and hospitalized patients with COVID-19, especially how both sides considered psychological support from each other.

## Methods

### Participants

The present study was approved by the research ethics committee of Peking University Health Science Center. Health-care workers and hospitalized patients with COVID-19 were recruited through an advertisement posted by doctors of the research team in a COVID-19 designated hospital in Wuhan from early February to late February 2020.

The health-care workers were eligible if they were working in the designated hospital during the study period. There were no specific exclusion criteria. The inclusion criteria for the patients with COVID-19 were the patients were diagnosed with COVID-19 and hospitalized in the same hospital. The patients who were too ill to fill in the online questionnaires independently were excluded.

Eligible participants were asked to scan a QR code and to complete the questionnaires online. All the participants were thanked and given 10RMB after finishing the questionnaires. Responses that were filled out in an unreasonable short period of time (within 3 min) were excluded. Finally, 311 health-care workers and 148 hospitalized patients with COVID-19 provided effective data for analyses.

## Measures

### Demographic Information

The health-care workers answered demographic questions such as gender, career, and professional title. In addition to the basic demographic information, the patients with COVID-19 were asked questions on the date of hospitalization, quarantine experience, a perceived pathway of infection, and the health conditions of family members.

### Psychological Symptoms

The Chinese version of the Self-reporting Questionaire-20 (SRQ-20) was used to measure neurotic symptoms such as depression, anxiety, and somatization. This validated tool consists of 20 questions requiring “yes” or “no” responses depending on the presence (scored as 1) or absence (scored as 0) of symptoms. The total score of the SRQ-20 has been reported to be associated with psychological symptoms with a good predictive effect for psychological disorders utilizing cutoff of scores higher than 7–8 (33). However, research in Chinese communities suggested total scores of 6–7 as a more appropriate cutoff (34). Thus, a cutoff of 6–7 was used in the present study. The Chinese version of SRQ-20 is considered reliable and valid (34).

To measure acute stress reaction (ASR) while reducing the burden to participants, 13 items were selected from the Stanford Acute Stress Reaction Questionnaire (SASRQ) (35). The Chinese version of SASRQ has been validated and has been repeatedly used to measure acute stress symptoms in Chinese samples (36). The selected items measured ASR in four domains, that is, the reexperience of traumatic events, high arousal and anxiety, disassociation, and avoidance. Some items were adapted to fit the specific context of the COVID-19 outbreak. For example, the original item “I had repeated distressing dreams of the event” had been changed as “I had repeated distressing dreams of what I witnessed in the hospital.” The items were rated on a 6-point Likert scale (0 = never, 5 = always), with a higher score indicating greater acute stress responses. The Cronbach's αs were 0.95 and 0.92 in the sample of health-care workers and patients, respectively.

### Psychological Stressors

Two scales were developed to measure the psychological stressors in the rapid growth stage of the COVID-19 outbreak. The items of the psychological stressors scale for the health-care workers were generated based on the literature of psychological stressors of health-care workers during the period of SARS outbreak (8, 10, 12). There were 11 items in the scale to measure the stressors of frontline health-care workers from four domains including infection fear (five items, for example, “I am worried about getting infected by COVID-19”), family concerns (two items, for example, “I feel guilty for letting my family worry about me”), discomfort caused by the precautionary measures (two items, for example, “protective gears cause physical discomfort”), and concerns about patients (two items, for example, “I worry about the deterioration of the condition of my patients”). Results of the exploratory factor analysis (EFA) indicated that all the items were grouped into one factor that explained 60.4% of the variance.

The version of the patients of the stressors scale was developed similarly based on the experience of patients during the SARS epidemic (15, 37, 38). There were 14 items measuring stress in four domains including survival threat, social stigma, loneliness, and guilt toward the family members. Results of EFA generated four factors that explained 66.8% of the variance. The factors were named as survival threat (five items, for example, “I feel that my life has been threatened.”), social stigma (four items, for example, “Everyone is avoiding me because of my disease”), loneliness and the feeling of losing control (three items, for example, “I feel lonely when staying in the quarantine ward”), and guilt about family (two items, for example, “I feel guilty for not being able to take care of my family”). The items were rated on a 5-point Likert scale (1 = totally disagree, 5 = agree). The average scores of each factor were involved in the analyses, with higher scores indicating greater perceived stress.

### Perceived External Coping Resources

Health-care workers and patients with COVID-19 were asked to select items that they thought helpful to cope with the stressors related to the coronavirus pandemic. For the frontline health-care workers, the external coping resources to be selected included social support from family members and friends (e.g., “keeping in touch with the family”), accessible psychological service (e.g., “using internet-based self-help psychological intervention program”), and organizational support (e.g., “the diagnosis and treatment standard and procedures have been established”).

For the patients who were hospitalized, medical staff support was included (e.g., “receiving greetings from doctors and nurses”) in addition to personal social support and accessible psychological service. To assess perceived supportive behaviors of health-care workers, patients were asked to select the behaviors that they thought were helpful including “encouragement,” “greeting,” “comforting,” “professional operation,” “smiling,” “telling the truth on the disease condition,” and “eye contact.”

### Statistical Analysis

Statistical product and service solutions (SPSS) 18.0 was used to analyze the data. Descriptive analyses were conducted to describe sample characteristics, stressors, psychological symptoms, and perceived external resources of participants. *T*-tests, ANOVA, and χ^2^-tests were used to compare demographic differences in psychological symptoms. EFA was used to explore constructs of pandemic-related psychological stressors for health-care workers and hospitalized patients. Correlations and multiple regressions were used to examine the associations between pandemic-related psychological stressors and psychological symptoms in both samples.

## Results

### Sample Demographics

The total sample size was 548, including 311 health-care workers and 148 hospitalized patients with COVID-19.

The sample of health-care workers was comprised of 217 (69.8%) local health-care workers and 94 (30.2%) medical team members who were deployed to Wuhan from other provinces of China^2^. There were 84 men (27%) and 227 women (73%). Seventy-nine participants were doctors (25.4%), 191 were nurses (61.4%), and 41 were medical technicians (13.2%). The age of the participants ranged from 17 to 60 years old, with an average age of 32 years old. The length of working experience, professional titles, and marital status of the health-care workers were displayed in Table 1 in detail.

**Table 1:** The demographic characteristics of participants.

	**The sample of frontline healthcare workers** **(*****n*** **=** **311)**		**The sample of patients with COVID-19** **(*****n*** **=** **148)**	
**Demographic characteristics**	**Frequency**	**Percentage (%)**	**Frequency**	**Percentage (%)**
Gender				
Male	84	27	82	55.4
Female	227	73	66	44.6
Age, yr	32.15 ± 7.09		40.51 ± 11.79	
<30	150	48.2	32	21.6
31–40	123	39.5	49	33.1
41–50	31	10.0	34	23.0
>51	6	1.9	33	22.3
Marital status				
Unmarried	95	30.5	20	13.5
Married	210	67.5	122	82.4
Divorced	5	1.6	5	3.4
Other	1	0.3	1	0.6
Affiliation				
The local hospital	217	69.8		
Medical teams	94	30.2		
Career				
Doctors	79	25.4		
Nurses	191	61.4		
Medical technicians	41	13.2		
Length of working experience, yr				
<5	101	32.5		
5–10	115	37.0		
>10	95	30.5		
Professional title				
Junior	168	54.0		
Intermediate	114	36.7		
Senior	29	9.3		
Place of residence				
Rural area			86	58.1
Urban area			62	41.9
Education attainment				
Junior high school and below			34	23.0
Senior high school			33	22.3
College			28	18.9
Bachelor degree			41	27.7
Master degree and above			12	8.1
Annual household income, RMB				
<30,000			29	19.6
30,000–80,000			40	27.0
80,000–120,000			30	20.3
120,000–200,000			26	17.6
200,000–300,000			17	20.3
300,000–1,000,000			5	3.4
>1,000,000			1	0.7

The sample of the hospitalized patients with COVID-19 contained 82 men (55.4%) and 66 women (44.6%). The average age was 40 years old (age range: 18–70). Eighty-six patients (58.1%) were from rural areas while 62 of them (41.9%) were from urban areas. Marriage status, educational attainment, and family income were exhibited in Table 1 in detail. In addition, 112 participants (75.7%) reported a quarantine experience before hospitalization while 36 of them (24.3%) had not been quarantined. Regarding the possible infection pathway, 23 (15.5%) thought they were infected by family members, 22 (14.9%) thought they were infected in the workplace, 50 (33.8%) considered they were infected in public places, and 44 (29.7%) could not ensure the pathway of infection. Among the patients, 70 (47.3%) reported that at least one of their family members was infected.

### Psychological Symptoms of Frontline Health-Care Workers and Hospitalized Patients With COVID-19

The means of neurotic symptoms and ASR of frontline health-care workers and patients with COVID-19 were exhibited in Table 2. For the frontline health-care workers, the mean of neurotic symptoms was 5.06 ± 4.78. Using 6/7 as the cutoff point, 104 out of 311 health-care workers (33.4%) scored 7 and above, which was indicative of a high risk of psychological disorders (34). The psychological symptoms of local health-care workers and medical team members deployed to Wuhan were compared. Results indicated that local health-care workers had a higher level of neurotic symptoms (5.61 ± 5.08) than medical team members deployed to Wuhan (3.79 ± 3.73), *t*_(236.83)_ = 3.53, *p* < 0.001, while the ratios of individuals with a high risk of psychological problems did not differ between the local health-care workers and the medical team members, ${\text{χ}}_{\left(1\right)}^{2}$ = 2.84, *p* = 0.092. The ASR scores were not significantly different between the local health-care workers and the medical team members as well, 15.19 ± 13.21 vs. 13.96 ± 14.12, *t*_(309)_ = 0.74, *p* = 0.459.

**Table 2:** Scores of SRQ-20 and ASR in frontline healthcare workers and patients with COVID-19.

	**Healthcare workers** **(*n* = 311)**	**Patients with COVID-19** **(*n* = 148)**
Neurotic symptoms	5.06 ± 4.78	5.49 ± 4.72
≤ 6	207	96
≥7	104	52
ASR	13.00 ± 11.68	14.73 ± 13.52
Re-experience	4.64 ± 3.64	3.72 ± 3.43
High arousal and anxiety	2.13 ± 2.08	3.70 ± 3.37
Disassociation	3.66 ± 3.93	3.78 ± 4.50
Avoidance	2.57 ± 3.10	3.53 ± 4.05

For hospitalized patients with COVID-19, the mean of neurotic symptoms was 5.49 ± 4.72. Fifty-two out of the 148 hospitalized patients with COVID-19 (35.1%) scored higher than seven on the SRQ-20. Health-care workers and patients were not significantly different in the scores of SRQ-20, *t*_(457)_ = −0.902, *p* = 0.368. The ratios of individuals with a high risk of psychological problems did not differ between the two samples, ${\text{χ}}_{\left(1\right)}^{2}$ = 0.128, *p* = 0.720.

### Pandemic-Related Psychological Stressors and Psychological Symptoms for Frontline Health-Care Workers and Patients With COVID-19

Correlations among pandemic-related psychological stressors of health-care workers and mental health indicators were displayed in Table 3. The total score of stressors was positively correlated with the neurotic symptoms (*r* = 0.35, *p* < 0.001) and the ASR (*r* = 0.38, *p* < 0.001). All the subscales of psychological stressors were significantly correlated with neurotic symptoms and the ASR, with the correlation coefficients ranging from 0.30 to 0.39.

**Table 3:** The means of pandemic-related psychological stressors of the healthcare workers and correlations with mental health (*n* = 311).

	**M ± SD**	**1**	**2**	**3**	**4**	**5**	**6**	**7**
1. Total scores of psychological stressors	13.35 ± 4.12	1						
2. Infection fear	3.31 ± 1.16	0.91**	1					
3. Family concerns	3.31 ± 1.20	0.88**	0.79**	1				
4. Concerns about patients	3.22 ± 1.11	0.88**	0.73**	0.68**	1			
5. Discomfort caused by the protective measures	3.51 ± 1.17	0.86**	0.69**	0.62**	0.69**	1		
6. Neurotic symptoms	5.06 ± 4.78	0.35**	0.35**	0.33**	0.30**	0.26**	1	
7. Acute stress responses	13.00 ± 11.68	0.38**	0.39**	0.35**	0.35**	0.25**	0.64**	1

**p < 0.01.

Hierarchical regression analyses were conducted to examine the effects of pandemic-related psychological stressors of health-care workers on their psychological symptoms after controlling gender, career, and affiliation that were significantly associated with neurotic symptoms and the ASR level. Because stressors of health-care workers in the four domains fell into one factor, the total score of stressors was used in the regression to avoid the problem of multicollinearity. As results indicated (see in **Table 5**), both models for neurotic symptoms and ASR were significant, with *F*_(5, 310)_ = 11.73, *p* < 0.01, *F*_(5, 310)_ = 10.85, *p* < 0.01, respectively. Pandemic-related psychological stressors positively predicted neurotic symptoms (β = 0.32, *p* < 0.001) and ASR (β = 0.36, *p* < 0.001).

The average scores of stressors for the patients with COVID-19 were displayed in Table 4. Results of the repeated measures ANOVA indicated that there were significant differences among the stress perceived in the four domains, *F*_(3, 441)_ = 65.06, *p* < 0.001. The score of guilt toward the family members was the highest, followed by survival threat. The scores of social stigma and loneliness and the feeling of losing control were the lowest and not significantly different from each other.

**Table 4:** The means of pandemic related stressors of the patients with COVID-19 and correlations with mental health status (*n* = 148).

	**M ± SD**	**1**	**2**	**3**	**4**	**5**	**6**
1. Survival threat	3.47 ± 1.00	1					
2. Social stigma	2.96 ± 1.07	0.48**	1				
3. Loneliness and the	2.74 ± 1.17	0.51**	0.44**	1			
feeling of losing control							
4. Guilt about family	3.96 ± 1.03	0.35**	0.37**	0.27**	1		
5. Neurotic symptoms	5.49 ± 4.72	0.42**	0.35**	0.50**	0.13	1	
6. Acute stress	14.73 ± 13.52	0.44**	0.43**	0.51**	0.15	0.64**	1
responses							

**p < 0.01.

Results of correlation analyses suggested that survival threat, social stigma as well as loneliness, and feeling of losing control were significantly correlated with neurotic symptoms and ASR level (*r*s ranged from 0.35 to 0.51), while the correlations of guilt toward the family members and neurotic symptoms and ASR were not significant.

Hierarchical regressions were further run to examine the effects of four psychological stressors on the neurotic symptoms and ASR of patients with gender, marital status, and health conditions of the families as covariates. Results indicated that the whole model was significant and explained a 31.3% variance in neurotic symptoms [*F*_(8, 146)_ = 9.30, *p* < 0.01]. Survival threat and loneliness and feeling of losing control significantly predicted an increase in neurotic symptoms, with β = 0.20, *p* = 0.025; β = 0.35, *p* < 0.001, respectively. However, the effects of social stigma and guilt toward family were not significant. The model was also significant to predict ASR [*F*_(8, 146)_ = 11.12, *p* < 0.01] and explained 34.7% variance in ASR. As demonstrated in Table 5, survival threat, loneliness and feeling of losing control, and social sigma had significant effects in predicting ASR for patients while the effect of guilt toward family was not significant.

**Table 5:** Regressions of pandemic-related psychological stressors on neurotic symptoms and ASR for frontline healthcare workers and patients with COVID-19.

**Sample**	**Dependent variable**	**Independent variable**	***R* ^**2**^**	**ΔR^**2**^Step 2**	**β**
Healthcare workers	Neurotic symptoms	Gender	0.15**	0.10**	0.03
		Career1			−0.10
		Career2			0.05
		Affiliation			−0.18**
		Stressors			0.32**
	ASR	Gender	0.14**	0.12**	0.01
		Career1			−0.04
		Career2			0.07
		Affiliation			−0.04
		Stressors			0.36**
Patients	Neurotic symptoms	Gender	0.31**	0.26**	0.17*
		Marriage1			0.03
		Marriage2			−0.08
		Health of family members			0.08
		Survival threat			0.20*
		Social stigma			0.09
		Loneliness and feeling of losing control			0.25**
		Guilt toward family			−0.01
	ASR	Gender	0.36**	0.29**	0.09
		Marriage1			0.10
		Marriage2			−0.06
		Health of family members			0.08
		Survival threat			0.18*
		Social stigma			0.20*
		Loneliness and feeling of losing control			0.33**
		Guilt toward family			−0.03

Career1 and Career2 were two dummy variables of career. Marriage1 and Marriage2 were two dummy variables of marital status. *p < 0.05, **p < 0.01.

### Perceived External Coping Resources for Frontline Health-Care Workers and Patients With COVID-19

Perceived external factors for the frontline health-care workers and patients with COVID-19 were listed by frequency in Table 6. The cooperation of patients was the most frequently selected (85.9%) for the health-care workers to cope with the pandemic. General support (e.g., “The hospital provides great support to the frontline health-care workers”) and professional support (e.g., “The diagnosis and treatment standard and procedure had been established”) from the hospital were also considered helpful by most of the participants. More than half of the perceived support of participants from family and friends was effective to cope with stressors related to the COVID-19 pandemic. Additionally, around 40% of health-care workers considered online psychological interventions were effective coping resources.

**Table 6:** Healthcare workers perceived external resources to cope with the coronavirus pandemic for the healthcare workers and the patients with COVID-19.

**External coping resources**	**Frequency of selection**	**Percentage (%)**
**For the frontline healthcare workers**		
1. The patients cooperate with the treatment nicely	267	85.9
2. The hospital provides great support to the front-line healthcare workers	254	81.7
3. The diagnosis and treatment standard and procedures had been established	253	81.4
4. Reading news about the cure rate increasing	252	81.0
5. Keeping in touch with the family	235	75.6
6. Receiving blessing from friends	203	65.3
7. Using internet-based self-help psychological intervention program	136	43.7
8. Using psychological relaxation program	130	41.8
**For the patients with COVID-19**		
1. Keeping in touch with the family	131	88.5
2. Receiving greeting from doctors and nurses	115	77.7
3. Reading news about the cure rate increasing	102	68.9
4. Receiving blessing from friends	90	60.8
5. Using internet-based self-help psychological intervention program	36	24.3
6. Using psychological relaxation program	31	20.9
**Perceived supportive behaviors from the healthcare workers**		
Encouragement	128	87.1
Greeting	124	84.4
Comforting	121	82.3
Professional procedures	117	79.6
Smiling	105	71.4
Telling the truth on the disease condition	89	60.5
Eye contact	67	45.6

For the patients with COVID-19, the most selected coping resource was “keeping in touch with the family” (88.5%). However, close to half (60.8%) considered support from friends was effective. The second most reported coping mechanism was support from medical staff (“receiving greetings from doctors and nurses,” 77.7%). The patients found emotional support in health-care workers through behaviors including encouragement, greeting, and comforting. Patients were found to feel supported through professional support such as professional procedures. The majority of patients (68.9%) reported being encouraged by the news reporting the progress of the treatment. Compared to health-care workers, fewer patients with COVID-19 perceived online psychological interventions as effective coping resources (20.9–24.3%).

## Discussions

The present study investigated psychological symptoms of the frontline health-care workers and the hospitalized patients in a designated hospital in Wuhan during the rapid growth stage of the COVID-19 pandemic (from early February to late February 2020), examined the effects of main psychological stressors on mental health in the two populations and identified perceived external coping resources for both sides. The results verified the important effect of reciprocal support between frontline health-care workers and patients in the COVID wards and had implications in providing integrated and specific psychological support for both sides.

### Psychological Responses of the Frontline Health-Care Workers and Patients With COVID-19

Both frontline health-care workers and patients with COVID-19 exhibited elevated psychological symptoms. We found 33.4% of health-care workers in the frontline combating the COVID-19 had significant psychological symptoms indicating a high risk for psychological disorders (for SRQ-20, 5.06 ± 4.78). The average score of SRQ-20 was higher compared with the finding in a previous study investigating the mental health of Chinese health-care workers in a nonepidemic period (41). This proportion of individuals at significant risk for psychological disorders was similar to prior studies focusing on the health-care worker treating patients with SARS (11, 42–45). The results were also largely in line with the findings of other studies that have investigated the mental health of the health-care workers in China during the current pandemic (2, 5). As demonstrated in our results, a significant number of frontline health-care workers showed no obvious psychological symptoms in the crisis but more than one-third of them experienced mental health disturbance in the rapid growth period of the COVID-19 outbreak. Because the acute psychological symptoms interfered with daily work and significantly predicted mental disorders such as depression, anxiety, posttraumatic disorder, and substance abuse (9, 44), it is important to provide psychological support to the high-risk individuals.

Regarding the psychological symptoms of hospitalized patients with COVID-19, 35.1% of them reported higher psychological symptoms and were identified as a high risk for psychological disorders. The prevalence was similar to the findings of the study of Kong et al. (46) focusing on the mental health status of patients with COVID-19 during hospitalization and other research investigating psychological symptoms of patients after recovery (19, 20). It is necessary to note that the clinical conditions of patients in the present study were mild to moderate. Patients with more severe symptoms might have more mental disturbance but they were not able to fill in the questionnaires by themselves. Therefore, the mental health problems for the hospitalized patients with COVID-19 might be underestimated in the present study.

### Reciprocal Support Might Facilitate Coping for Both Health-Care Workers and Patients

As indicated in the results, concern about patients is an aspect of psychological stressors for health-care workers. Health-care workers worried about the clinical conditions and emotional reactions of the patients. This is in line with the observations of Chen et al. (47). According to their report, health-care workers often did not know how to deal with the situation when the patients refused to cooperate with the infection control measures or exhibited strong negative emotional reactions. Therefore, strategies reducing stress responses of patients or facilitating their cooperation would be effective to reduce the psychological reactions of frontline health-care workers.

Consistent with this, it has been found that the cooperation of patients was the most selected effective coping resource for health-care workers. We agreed with Chen et al. (47) that it is necessary to provide training and counseling to the health-care workers on how to communicate with patients on the infection control measures and general skills to deal with the psychological symptoms of the patients.

For hospitalized patients with COVID-19, survival threat, loneliness and the feeling of losing control, and social stigma were significantly associated with psychological symptoms after controlling related covariates. Treatment, health education, and encouragement from the health-care workers were vital to reducing the psychological stressors of patients.

Regarding the perceived coping resources, family support was the most selected item that is effective to improve the psychological health of the patients with COVID-19. This is in line with previous findings (26). However, when staying in the quarantined wards in the hospital, communication with family members was relatively indirect. Thus, it is important to encourage the patients to contact their families or friends through phone or the internet. For the patients who could not use the devices, it would be beneficial if the nurses help them to do that. Apart from a personal social support network, the support from the health-care workers was perceived as important. Encouragement from the medical staff and professional procedures might be effective to reduce the survival threat of the patients. Simple communications such as greeting and eye contact were also supportive to the patients.

In summary, the influences between health-care workers and the patients have been observed. First, reducing stress responses of the patients might be effective to reduce psychological stressors of the frontline health-care workers and in turn promote their mental health status. Second, health-care workers played an important role in reducing psychological stressors of patients such as survival threat, social stigma and loneliness, and the feeling of losing control. Third, both health-care workers and patients with COVID-19 perceived support from the opposite side as a helpful external coping resource in dealing with the COVID-19 crisis.

## Limitations

There are several limitations to the present study. First, convenience sampling was used in the present study and the sample size was relatively small. The majority of participants in the health-care workers were female aged below 40 and with the junior to the intermediate professional title. The patients had relatively mild to moderate clinical conditions. Because of the limitations in sample size and representativeness of the samples, the results had limited generalizability especially to populations that are very different in the demographic characteristics from our samples. Second, items of psychological stressors of the frontline health-care workers and hospitalized patients with COVID-19 were generated from findings of previous studies in the SARS outbreak. The frontline health-care workers and patients with COVID-19 were not given the opportunity to express their experiences through qualitative studies. Thus, the situation-specific stressors in this pandemic might be neglected. Third, although the perceived external coping resources were identified, their effects to reduce psychological symptoms for the frontline health-care workers and the patients with COVID-19 in the acute phase of disease were not empirically examined. This topic should be examined using a longitudinal design in future studies.

## Implications

Nevertheless, the present study has practical implications in the psychological interventions for the frontline health-care workers combatting COVID-19 and hospitalized patients with COVID-19. Our findings supported that a considerable proportion of the health-care workers and the patients with COVID-19 had significant psychological symptoms that need psychological support. Strategies to reduce the stressors of health-care workers and the patients might be effective. In addition to the individual social support system, our findings emphasize the importance of treatment alliance in reducing psychological symptoms for both medical staff and the patients with COVID-19. Supportive behaviors of health-care workers might be effective to reduce the stress of the patients and in turn, reduce the stress of the health-care workers themselves.

## Data Availability Statement

The raw data supporting the conclusions of this article will be made available by the authors, without undue reservation.

## Ethics Statement

The studies involving human participants were reviewed and approved by research Ethics Committee of Peking University Health Science Center. Written informed consent for participation was not required for this study in accordance with the national legislation and the institutional requirements.

## Author Contributions

TZ and RG: conceptualization. TZ: methodology, analysis, and writing—original draft preparation. LS: investigation. RG, ZG, SR, and SM: writing—review and editing. RG and TZ: funding acquisition. All authors have read and agreed to the published version of the manuscript.

## Funding

This work was supported by the Fundamental Research Funds for the Central Universities (Grant Number: BMU2021RCZX003), Special Research Fund of PKU for Prevention and Control of COVID-19, and Chen Zhong-geng Foundation for the Professional Development of Clinical and Counseling Psychology (Grant Number: 2020YJ-JT001).

## Conflict of Interest

The authors declare that the research was conducted in the absence of any commercial or financial relationships that could be construed as a potential conflict of interest.

## Publisher's Note

All claims expressed in this article are solely those of the authors and do not necessarily represent those of their affiliated organizations, or those of the publisher, the editors and the reviewers. Any product that may be evaluated in this article, or claim that may be made by its manufacturer, is not guaranteed or endorsed by the publisher.

## Footnotes

^1^Wuhan was the first city where the COVID-19 appeared in China, and it reached its peak of newly confirmed cases during the period of January to February 2020 (30–32).

^2^To meet the urgent needs of medical equipment and health-care workers, the Chinese government mobilized medical forces from other provinces in China. By March 8, 2020, 346 medical teams had been sent from major hospitals in China, with 42,600 health-care workers in total to support Wuhan and other areas of Hubei Province. These health-care workers were mainly physicians and nurses from departments of respiratory medicine and intensive care units, as well as health-care workers from subspecialties such as infectious diseases, cardiology, nephrology, and psychology (39, 40).
